# Dirac cone protected by non-symmorphic symmetry and three-dimensional Dirac line node in ZrSiS

**DOI:** 10.1038/ncomms11696

**Published:** 2016-05-31

**Authors:** Leslie M. Schoop, Mazhar N. Ali, Carola Straßer, Andreas Topp, Andrei Varykhalov, Dmitry Marchenko, Viola Duppel, Stuart S. P. Parkin, Bettina V. Lotsch, Christian R. Ast

**Affiliations:** 1 Max Planck Institute for Solid State Research, Heisenbergstr. 1, 70569 Stuttgart, Germany; 2 Max Plank Institute for Microstructure Physics, Weinberg 2, 06120 Halle, Germany; 3 IBM-Almaden Research Center, 650 Harry Road, San Jose, California 95120, USA; 4 Helmholtz-Zentrum Berlin für Materialien und Energie, Elektronenspeicherring BESSY II, Albert-Einstein-Straße 15, 12489 Berlin, Germany; 5 Department of Chemistry, Ludwig-Maximilians-Universität München, Butenandtstr. 5-13, 81377 München, Germany; 6 Nanosystems Initiative Munich (NIM) & Center for Nanoscience, Schellingstr. 4, 80799 München, Germany

## Abstract

Materials harbouring exotic quasiparticles, such as massless Dirac and Weyl fermions, have garnered much attention from physics and material science communities due to their exceptional physical properties such as ultra-high mobility and extremely large magnetoresistances. Here, we show that the highly stable, non-toxic and earth-abundant material, ZrSiS, has an electronic band structure that hosts several Dirac cones that form a Fermi surface with a diamond-shaped line of Dirac nodes. We also show that the square Si lattice in ZrSiS is an excellent template for realizing new types of two-dimensional Dirac cones recently predicted by Young and Kane. Finally, we find that the energy range of the linearly dispersed bands is as high as 2 eV above and below the Fermi level; much larger than of other known Dirac materials. This makes ZrSiS a very promising candidate to study Dirac electrons, as well as the properties of lines of Dirac nodes.

## Abstract (web summary)

The family of topological materials has been growing rapidly but most members bare limitations hindering the study of exotic behaviour of topological particles. Here, Schoop *et al*. report a Fermi surface with a diamond-shaped line of Dirac nodes in ZrSiS, providing a promising candidate for studying two-dimensional Dirac fermions.

## Graphical abstract

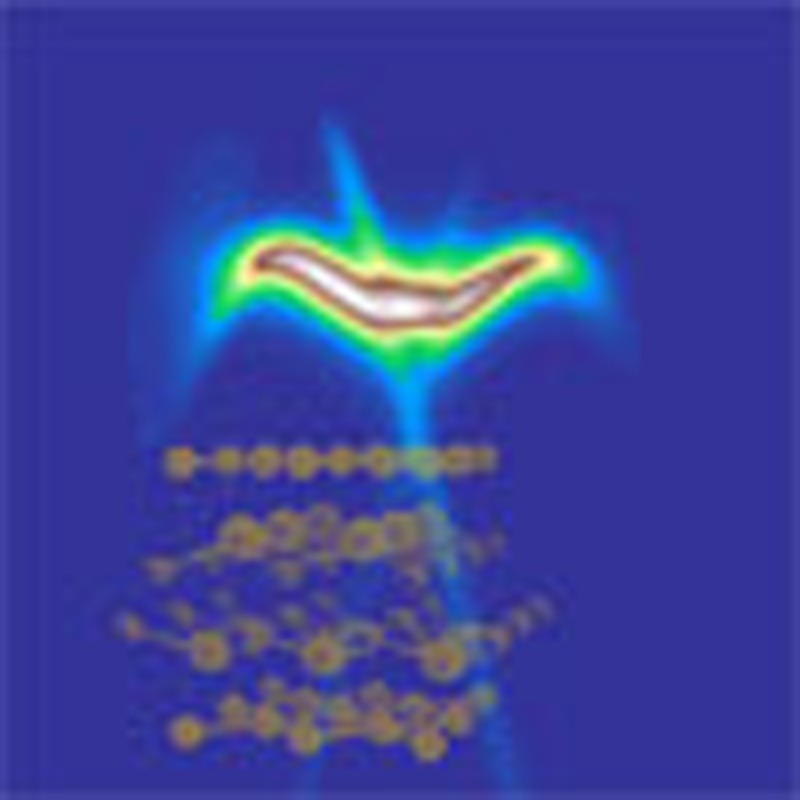

The electronic structure of a three-dimensional (3D) Dirac semimetal (DSM) contains two sets of linear, doubly degenerate bands which cross at a fourfold degenerate crossing called a Dirac point, a sort of 3D analogue of graphene^1^,^2^. If inversion symmetry or time reversal symmetry are broken, those doubly degenerate bands become spin split, resulting in singly degenerate band crossings called Weyl nodes^3^,^4^. Although many different materials have been predicted to host Dirac or Weyl fermions^2^,^5^,^6^, only a few real materials have been experimentally verified. Both Cd_3_As_2_ and Na_3_Bi have symmetry protected 3D Dirac cones, which have been imaged with angle-resolved photoelectron spectroscopy (ARPES)^7^,^8^,^9^,^10^. Both materials exhibit exotic transport properties such as ultra-high mobility, large, linear magnetoresistance (MR) and negative magnetoresistance at low fields^11^,^12^,^13^,^14^,^15^. Signatures of a chiral anomaly in ZrTe_5_ have been seen in ARPES as well as transport experiments^16^. Weyl fermions have been shown to exist in the inversion symmetry-breaking compounds TaAs (ref. 17, 18), NbAs (ref. 19) and TaP (ref. 10; and predicted in WTe_2_, MoTe_2_ and other Ta or Nb monopnictides^5^,^20^,^21^). Very recently, Weyl nodes were shown in the intrinsically time reversal symmetry-breaking compound, YbMnBi_2_ (ref. 22). Young and Kane used the concept of non-symmorphic symmetry to predict that in two-dimensional (2D) square lattices, new types of 2D DSMs can exist that are distinct from both graphene and 3D DSMs (ref. 23). In particular, these 2D Dirac cones may host 2D Dirac fermions with a behaviour distinct from their 3D analogues; experimental verification is pending. Most interestingly, Young and Kane showed that those 2D Dirac cones protected by non-symmorphic symmetry cannot be gapped by spin–orbit coupling (SOC), in contrast to 2D cones protected by different symmetries. Finally, materials with Dirac line nodes, where the Fermi surface forms a closed loop, have recently been predicted but only experimentally verified in one material, PbTaSe_2_, where other bands are interfering at the Fermi level^24^,^25^,^26^,^27^,^28^,^29^. Those materials have been predicted to have exotic physical properties such as surface magnetism or superconductivity^30^ as well as long-range Coulomb interactions^31^. Furthermore, a flat, non-dispersive Landau level has been proposed to exist in these materials^27^.

In all of the currently known DSMs, the energy range of the linear dispersion of the Dirac cone is very small. In Cd_3_As_2_, the Lifshitz transition appears according to calculations only ∼20 meV above the Fermi level. In the real material, however, the Fermi energy has been shown to lie 200 meV above the Dirac cone^8^. In Na_3_Bi, TaAs and other monopnictides, the Lifshitz transition is only roughly 100 meV away from crossings. A material with a larger linear dispersion would allow easy study of Dirac and Weyl physics despite changes in the Fermi level due to defects or impurities. The fabrication of thin films and devices from Dirac and Weyl materials would greatly benefit from more robust Dirac and Weyl states due to the difficulties in achieving thin film quality approaching that of single crystals. Also, many of the known materials have further disadvantages, such as the toxicity of arsenides as well as the extreme air sensitivity and chemical instability of Na_3_Bi, which also make studying their exotic physics difficult.

Here, we show by electronic structure calculations and ARPES that the so far unnoticed system, ZrSiS, exhibits several Dirac crossings within the Brillouin zone which form a diamond shaped Fermi surface with a line of Dirac nodes, without any interference of other bands. This compound is non-toxic and highly stable with band dispersions of the linear region of those crossings being larger than in any other known compound: up to 2 eV in some regions of the Brillouin zone. The range in which all bands are linearly dispersed is roughly 0.5 eV. SOC introduces a small gap to the Dirac cones near the Fermi surface, of the size of ≈20 meV (much less than in the related Bi-based compounds). We also show the presence of a Dirac feature below the Fermi level, which is generated by the square Si sublattice and is protected by the non-symmorphic symmetry through a glide plane (regardless of SOC strength), supporting the recent prediction by Young and Kane regarding 2D Dirac fermions^23^. We also show that around this Dirac feature an unusual, previously not predicted surface-derived state arises. Thus, ZrSiS is a very promising candidate for investigating Dirac and Weyl physics, as well as the properties of lines of Dirac nodes.

## Results

### Crystal structure

ZrSiS crystallizes in the PbFCl structure type in the tetragonal *P4/nmm* space group (no. 129) (refs 32, 33). It is related to the Weyl semimetal, YbMnBi_2_, whose structure is a stuffed version of the PbFCl structure type, hence the different stoichiometry. Other Bi based, stuffed PbFCl structures, such as EuMnBi_2_ and (Ca/Sr)MnBi_2_, have also already been shown to host Dirac electrons and to exhibit exotic transport properties^34^,^35^,^36^. Both structures display square nets of Si and Bi atoms, respectively, that are located on a glide plane.

**Figure 1:**
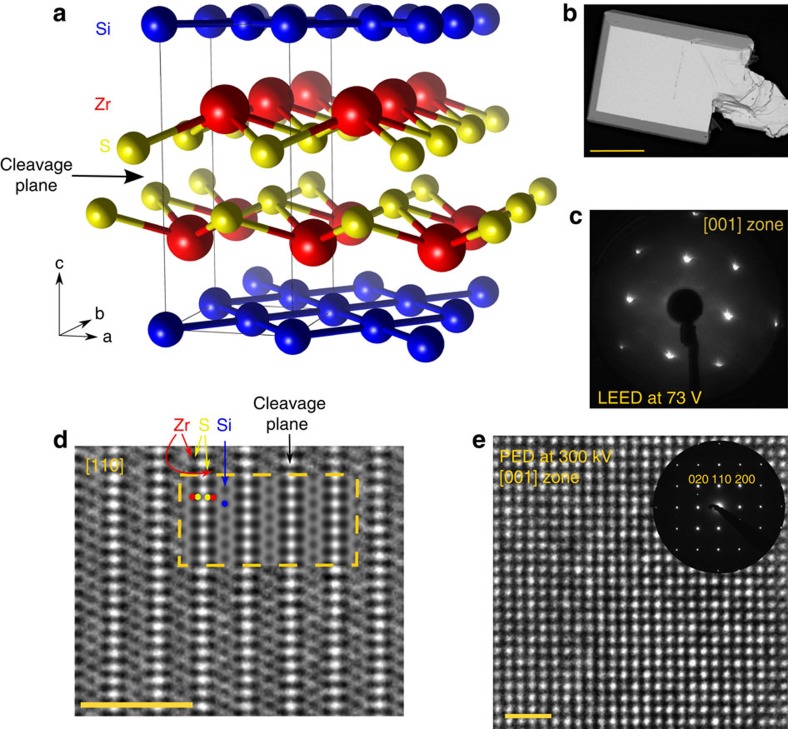
Structure and characterization of ZrSiS single crystals. (**a**) Crystal structure of ZrSiS. The Si square net can be seen in blue. (**b**) SEM image of a typical crystal. The scale bar corresponds to 0.5 mm. (**c**) Low-energy electron diffraction (LEED) pattern of a cleaved crystal showing that it cleaves perpendicular to the *c* axis. (**d**) HRTEM image of the [110] orientation, inset shows simulated HRTEM image. The scale bar corresponds to 2 nm. The focus plane is Δ*f*=−50 nm, close to the Scherzer focus, where atoms appear in black. Individual atoms could be identified and the cleavage plane between sulfur atoms is visible in white. For images with different foci and their simulations see Supplementary Fig. 2. (**e**) HRTEM image and PED pattern of the (001) surface, the square arrangement of atoms is clearly visible. The scale bar corresponds to 1 nm.

The crystal structure of ZrSiS is displayed in Fig. 1a (see Supplementary Tables 1 and 2 for crystallographic information). The Si square net is located in the *ab* plane and layers of Zr and S are sandwiched between the Si square nets in such a way that there are neighbouring S layers in between the Zr layers. We were able to image the square lattices with high-resolution transmission electron microscopy (HRTEM) shown in Fig. 1d,e (see Supplementary Figs 1 and 2 for more information on precession electron diffraction (PED) and HRTEM, respectively). The HRTEM image of the (110) surface shows a gap between neighbouring S atoms, which is where the crystals cleave. The highly ordered structure in the HRTEM images as well as the very good *R*_Bragg_ value for the single-crystal refinement indicate extremely high sample quality. The low-energy electron diffraction pattern shown in Fig. 1c clearly indicates a square arrangement of Bragg reflections, hence showing that the crystals cleave perpendicular to the tetragonal *c* axis. There is no sign of a surface reconstruction happening in these crystals. A scanning electron microscopy (SEM) image of a typical crystal is shown in Fig. 1b. The crystals are very stable in water and air, and only dissolve in concentrated acids.

### Calculated electronic structure

**Figure 2:**
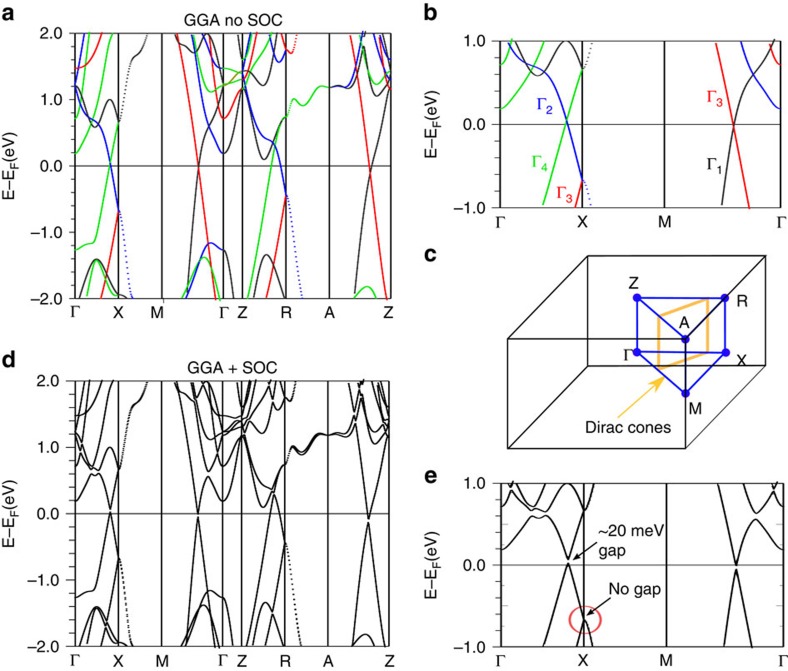
Calculated bulk band structure of ZrSiS. (**a**) Bulk band structure without SOC, each colour of the line represents a different irreducible representation; they are labelled in the closed-up image (**b**). (**c**) Brillouin zone of a primitive tetragonal lattice; the Dirac cone line nodes along the symmetry lines are labelled in orange. (**d**) Bulk band structure with SOC; a very small gap is opened but the overall change to the band structure remains small. In the zoomed in panel (**e**) the cone at X, which is protected by non-symmorphic symmetry, is circled in red.

The calculated electronic structure of bulk ZrSiS is displayed in Fig. 2. Without SOC, several Dirac cones are visible which cross along ΓX and ΓM as well as along ZR and ZA, which are the respective symmetry lines above ΓX and ΓM. However, the crossing along ZR is higher in energy compared with the one along ΓX. The cones are protected by the *C*_2*v*_ symmetry along those lines. The protection is similar to the one in the 3D line node material Ca_3_P_2_ (refs 25, 30). The Dirac cones form an unusually shaped line node in the Brillouin zone, displayed in Fig. 2c (this schematic assumes all Dirac points to be at the same energy to make it easier to understand the principle of the electronic structure). This gives rise to a diamond shaped Fermi surface. Note that the range in which the bands are linearly dispersed is very large compared with other known Dirac materials, although it should be pointed out that nonlinear bands interfere slightly above the Fermi level. Nevertheless, there is still an energy window of roughly 0.5 eV in which the Fermi energy only cuts through linearly dispersing bands. The electronic structure without SOC is very similar to that observed in YbMnBi_2_; however, since the symmetry along the lines with the Dirac crossings is *C*_2*v*_, SOC gaps the cones. The *C*_2*v*_ point group allows for only a single irreducible representation in the presence of SOC. In Bi-based compounds, the effect of SOC is very dramatic. It also destroys the large dispersion of the linear bands. In ZrSiS, however, SOC is small and only produces very small gaps (roughly 20 meV) in the cones along the *C*_2*v*_ symmetry lines, maintaining the large linear dispersion (Fig. 2). Furthermore, in Bi-based compounds, more bands interfere with the cone structure around the Fermi energy.

There are other Dirac-like crossings at the X and R point that are located at −0.7 and −0.5 eV, respectively. These crossings are protected by the non-symmorphic symmetry of the space group, very similar to the recently predicted Dirac cones in 2D square nets and are not influenced by SOC (ref. 23). The lack of an effect of SOC to these types of cones was predicted by Young and Kane but can also be seen in our electronic structure calculation. In this template system, along the XM direction, both bands fold on the same energy. Young and Kane predicted exactly this electronic structure for a square net system, a structural motif appearing in ZrSiS. They predicted several scenarios to lift the degeneracy along XM subsequently to host 2D Dirac fermions. To the best of our knowledge this is the first time such a feature in the electronic structure has been observed in a real material. This Dirac-like crossing is significantly below the Fermi level with other bands also present, however, hole doping (on the Zr or S site) or the gating of thin films may allow for investigation of the physics of the 2D Dirac fermions.

### Angle-resolved photoemission measurements

**Figure 3:**
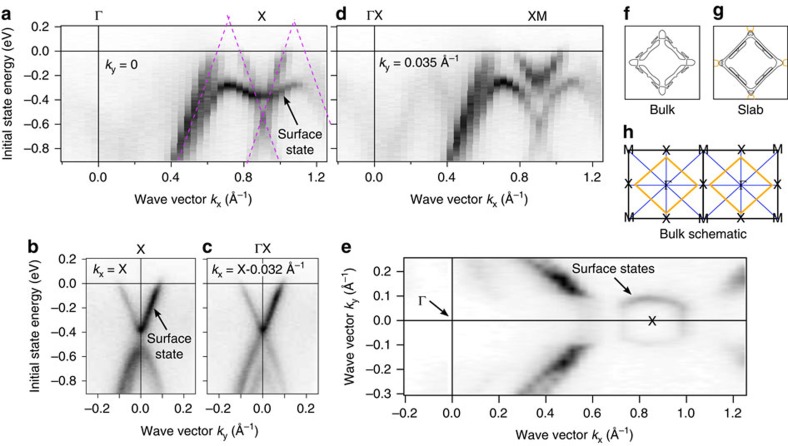
Band structure measured with ARPES. (**a**) Band structure along ΓX. The purple lines represent the predicted bulk bands. In addition a surface state is visible. (**b**) Band structure along MXM and parallel to MXM (**c**). Along the high symmetry line the band structure is gapped (**b**) but with a much smaller gap than predicted in the bulk calculation. The gap closes and a cone forms parallel to the high symmetry line (**c**). (**d**) Band structure parallel to ΓX. Due to the gapping of the surface state it can be inferred that it is hybridized with the bulk cone at X. (**e**) Constant energy plot at the Fermi energy. (**f**–**h**) The lower drawing (**h**) sketches the predicted Fermi surface and compares calculated bulk and slab Fermi surfaces (**f**,**g**). Pockets that are clearly surface-derived are drawn in orange. The measured Fermi surface displays the predicted slab Fermi surface well.

**Figure 4:**
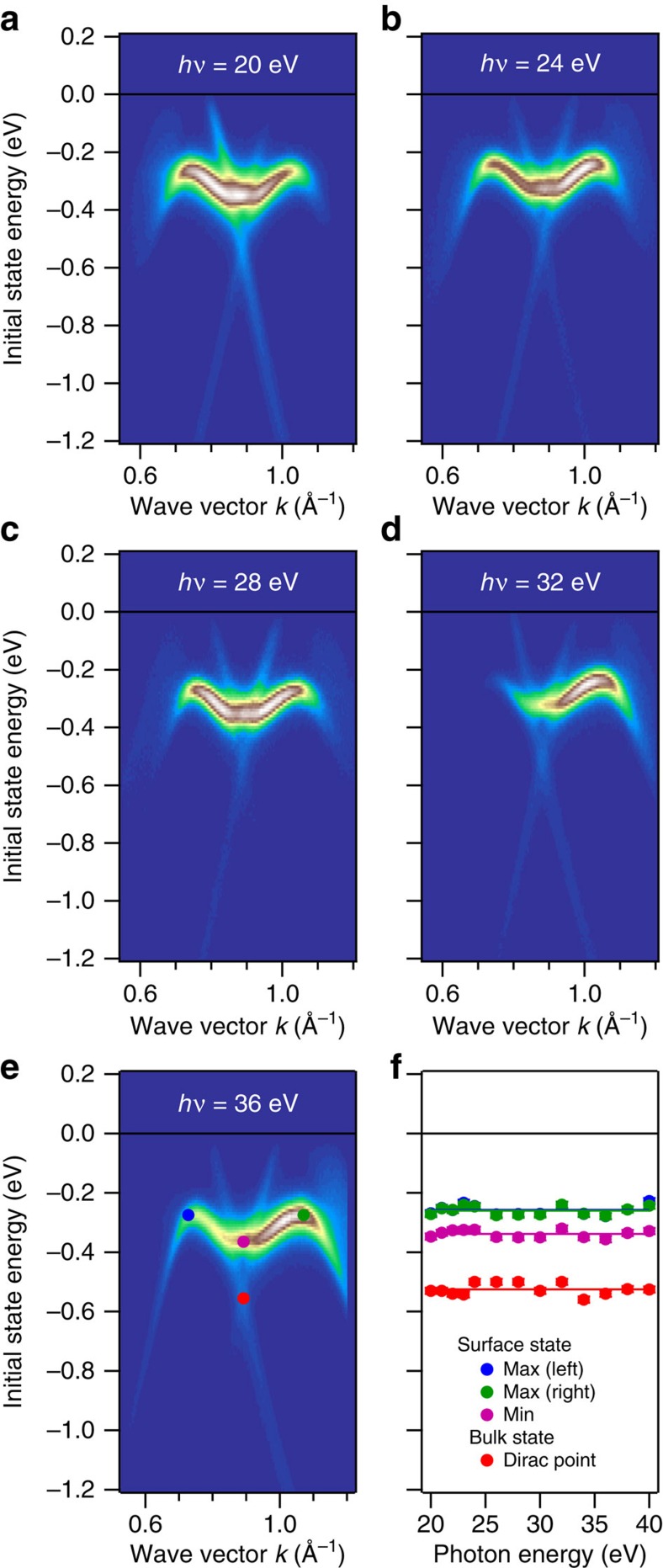
ARPES spectra of ZrSiS near X along ΓX at different photon energies between 20 and 40 eV. Selected photon energies are shown in panels (**a**–**e**). The position of the bands does not change, as shown in panel (**f**) for selected points in the band structure, supporting the 2D character of the states. Changes in intensity are due to changing matrix elements as a function of photon energy.

ARPES data are shown in Fig. 3. The cone protected by non-symmorphic symmetry at X is clearly visible at −0.5 eV (Fig. 3a). Perpendicular to ΓX, both bands fold on the same energy along the XM direction (Fig. 3b) exactly matching the prediction of Young and Kane^23^. The Dirac points of the cones along the ΓX line in Fig. 3a are not completely visible since the Fermi level is slightly below the Dirac points (as also predicted in the slab calculation shown below). Dashed purple lines indicate the predicted bulk bands. Note that we observe linearly dispersed bands for >1 eV energy range below the Fermi level, as predicted by the calculation. We find the Fermi velocity of these bands to be *ħv*_F_=4.3 eV Å, which is only slightly lower than the Fermi velocity in graphene (*ħv*_F_=6.7 eV Å) (ref. 37). This indicates that in spite of the small gap created by SOC, the electrons should still have a very low mass. We observe additional states along ΓX (Fig. 3a) not seen in the calculated bulk band structure, which we attribute to surface-derived states. This is supported by slab calculations shown below and by data taken at more photon energies shown Fig. 4 (see Supplementary Figs 3 and 4 for more details). As can be seen in Fig. 4, the band dispersion close to the X point does not change with varying the photon energy clearly indicating that the states in question are 2D. When moving parallel to ΓX towards XM (Fig. 3d), one can see how this surface-derived state interacts with the bulk bands near X. This hybridization of the alleged surface state with the conical bulk state near the X point may be attributed to the inherent 2D character of this bulk state. Normally, a surface state does not exist within the projected bulk band structure. However, as the bulk state is rather 2D itself, we surmise that the bands hybridize in the vicinity of the surface. Since ARPES is an extremely surface-sensitive technique and the unit cell along the *c* axis is rather long, the actual bulk band dispersion inside the crystal remains unobservable. Figure 3b shows the measured band structure along MXM. Along the high symmetry line, a gap is observed, which is much smaller than the gap in the bulk band structure, due to the presence of surface-derived states along this direction as well. Another cone at −0.4 eV is visible in the measurement slightly parallel to the high symmetry line, towards the ΓX direction (Fig. 3c). This cone is not seen in the bulk band structure calculation (Supplementary Fig. 5), which indicates that this Dirac cone is also surface-derived. It is connected to the surface-derived state along ΓX as seen in Fig. 3a as well as in the slab calculation (see below). In Fig. 3e, we show a constant energy plot of the Fermi level. From the bulk calculation, we expect a diamond shaped Fermi surface as sketched in Fig. 3h. In the experimental data, we not only observe this diamond shaped Fermi surface, but, in addition, the data shows the surface-derived state around X to cross the Fermi level as well. The calculated slab Fermi surface in Fig. 3g is in excellent agreement with the measured Fermi surface. If a constant energy plot is taken at lower energies (Supplementary Fig. 6) the observed ARPES spectrum matches very well with the predicted constant energy surface at −515 meV of the bulk surface, due to the absence of surface states at this energy.

## Discussion

**Figure 5:**
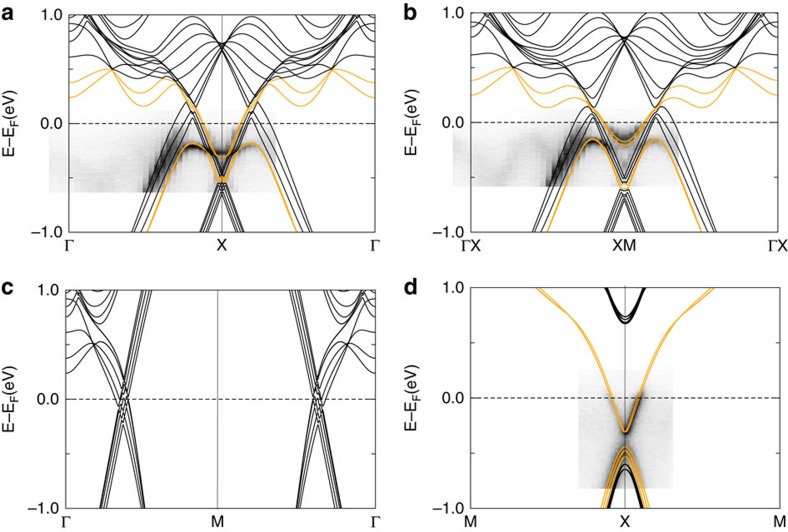
Calculated slab band structure in comparison with the measured band structure. (**a**) Bands along ΓX. The orange bands indicate how bands are progressing, showing the hybridization of the surface-derived state with the bulk. (**b**) Bands parallel to ΓX highlighting the mixing of surface-derived states and bulk bands belonging to the cone at X. (**c**) Slab band structure along ΓM, showing that there is no big change to the bulk band structure in this direction. (**d**) Bands along MXM, surface-derived bands are highlighted in orange. The surface states significantly reduce the bulk gap along this direction.

To ascertain the nature of the alleged surface states, we performed band structure calculations of a slab. The resulting band structures in comparison to ARPES data are shown in Fig. 5. SOC is included for these calculations. The creation of a surface causes several changes to the electronic structure as compared with the bulk calculation; the cone along ΓM remains unchanged (Fig. 5c) but the cone along ΓX moves up in energy compared with the bulk structure. The same is true for the cone at X (Fig. 5a). This can be understood if one considers that in a 2D slab, the ZR bands are projected onto the ΓX bands. In addition, the surface-derived state seen in ARPES appears along ΓX. The high level of agreement between the predicted and measured electronic structure is shown by superimposing the two images in the figure without rescaling. Considering the combination of bulk and slab calculations, we conclude that the states in question are surface-derived. This is further strengthened by the data taken at different photon energies (between 20 and 40 eV), which shows no change in the band dispersion (see Fig. 4 as well as Supplementary Figs 3 and 4). Furthermore, the measured Fermi energy matches the predicted one; however, the ARPES data are measured at room temperature which indicates that the samples are slightly hole doped.

Continuous bands along this surface-derived state are highlighted in orange in Fig. 5a. These bands do not follow the expected path of the surface state, thus also indicating the hybridization with the bulk. A simulation of the slab band structure parallel to ΓX is shown in Fig. 5b. In accordance with ARPES the bands that form the surface state split apart. Calculations of the slab without SOC (Supplementary Fig. 7a) show that the bands forming the surface-derived state and the cone around X have different irreducible representations in the absence of SOC, along ΓX, but not parallel to it (Supplementary Fig. 7b). This indicates that SOC cannot be held responsible for the hybridization. Supplementary Fig. 7c shows the contribution of the surface atoms to the slab band structure. This supports the surface character of the additional band observed in the experiment. Although the surface-derived states seem to hybridize with the bulk bands, this scenario is only expected to appear locally and in close vicinity to the crystal surface. It should therefore not affect the bulk properties of this material where high carrier mobilities and high magnetoresistance is expected to be seen in a transport experiment.

Figure 5d shows the predicted slab band structure along XM. Surface-derived states that lie in between the bulk bandgap in this part of the Brillouin zone are highlighted in orange. The surface-derived states are mainly appearing around the X point. Again, ARPES data matches well with the prediction, the observed decreasing gap along this direction is also seen in the slab calculation showing that this is caused by the surface.

In summary, we showed that ZrSiS, a stable and non-toxic material, has a very exotic electronic structure with many Dirac cones that form a diamond shaped Fermi surface. The bands are linearly dispersed over a very large energy range, larger than in any other material reported to date. The non-zero, but small SOC creates a gap of ∼20 meV in the Dirac cones close to the Fermi level. We confirmed our electronic structure calculations with ARPES measurements that are in excellent agreement with the calculated structure. We also show the first experimental realization of a template system for 2D Dirac cones protected by non-symmorphic symmetry, in excellent agreement with recent theoretical predictions; the band structure we observe in ZrSiS at 0.5 eV below the Fermi level is very similar to the prediction of the band structure of a hypothetical square lattice. In addition, we observe an unconventional surface-derived state that is hybridized with bulk bands around the X point. It is uncommon for a surface state to exist within the projected bulk band structure or even hybridize with it. A possible cause might be the 2D nature of the bulk bands around X; however, further investigation into this effect is required. In contrast to compounds with a Bi square net, where a large SOC opens a large gap with parabolic dispersion, ZrSiS has a Si square net where the SOC effect is very much reduced and linear dispersion of the bands is mostly preserved. Since no other bands interfere at the Fermi level, the unusual electronic structure of ZrSiS makes it a strong candidate for further studies into Dirac physics; especially magnetotransport, since the Fermi energy can be tuned quite substantially, while still being in the linear range of the bands.

*Note added in proof*: during the review process the following papers related to our work were submitted to arXiv (refs 38, 39, 40, 41, 42). We find that the ARPES results presented in these reports are well in line with our results.

## Methods

### Sample synthesis and characterization

Single crystals of ZrSiS were grown in a two-step synthesis. First, a polycrystalline powder was obtained following the same procedure as in ref. 32. In a second step single crystals were grown from the polycrystalline powder via I_2_ vapour transport at very high quality 1,100 °C with a 100 °C temperature gradient. The crystals were obtained at the cold end. The published crystal structure was confirmed with single-crystal x-ray diffraction and electron diffraction. The crystal used for single-crystal x-ray diffractiion (SXRD) was of very high quality and an *R*_1_ value of 1.5% was obtained for the structural solution (see Tables S1 and S2 in the Supplemental Information for more details). SXRD data were collected on a STOE IPDS II working with graphite monochromated Mo K_*α*_ radiation. Reflections were integrated with the STOE X -Areaa 1.56 software and the structure was solved and refined by least squares fitting using SHELXTL (ref. 43). Electron microscopy was performed with a Phillips CM30 ST (300 kV, LaB_6_ cathode). HRTEM images and PED patterns were recorded with a complementary metal–oxide–semiconductor (CMOS) camera (TemCam-F216, TVIPS); the microscope is equipped with a nanoMEGAS spinning star to obtain PED images. The programme JEMS (Stadelmann) was used to simulate diffraction patterns and HRTEM micrographs.

### Angle-resolved photoemission

For ARPES measurements crystals were cleaved and measured in ultra-high vacuum (low 10^−10^ mbar range). Low-energy electron diffraction showed that the cleavage plane was the (001) plane. ARPES spectra were recorded at room temperature with a hemispherical PHOIBOS 150 electron analyzer (energy and angular resolution are 15 meV and 0.5°, respectively). As photon source a monochromatized He lamp that offers ultraviolet radiation at *hν*=21.2 eV (He I). In addition, photon-energy-dependent measurements between *hν*=20 and 40 eV were done at room temperature with the 1^2^-ARPES experiment installed at the beamline UE112-PGM2a at BESSY-II. SEM images of crystals were measured with a SEM (Vega TS 5130 MM, Tescan) using a Si/Li detector (Oxford).

### Electronic structure calculations

Electronic structure calculations were performed in the framework of density functional theory using the WIEN2K (ref. 44) code with a full-potential linearized augmented plane-wave and local orbitals [FP-LAPW+lo] basis^45^ together with the Perdew–Becke–Ernzerhof parameterization^46^ of the generalized gradient approximation as the exchange-correlation functional. The plane-wave cutoff parameter R_MT_K_MAX_ was set to 7 and the irreducible Brillouin zone was sampled by 1,368 k-points (bulk) and by a 30 × 30 × 3 mesh of k-points (slab). Experimental lattice parameters from the single-crystal diffraction studies were used in the calculations. SOC was included as a second variational procedure. For the slab calculation it was found that cleaving between sulfur atoms resulted in the closest match to the experimental observation. This cleavage plane is in agreement with HRTEM imaging and chemical intuition. The slab was constructed by stacking 5 unit cells in *c* direction that were gapped by a 5.3 Å vacuum.

### Data availability

The data that support the findings of this study are available from the corresponding author upon request.

## Additional information

**How to cite this article:** Schoop, L. M. *et al*. Dirac cone protected by non-symmorphic symmetry and three-dimensional Dirac line node in ZrSiS. *Nat. Commun.* 7:11696 doi: 10.1038/ncomms11696 (2016).

## Supplementary Material

Supplementary Information Supplementary Figures 1-7 and Supplementary Table 1-2
