# Supplementary material for: Dirac cone protected by non-symmorphic symmetry and three-dimensional Dirac line node in ZrSiS
Source: Nat Commun. 2016 May 31;7:11696. doi: 10.1038/ncomms11696 (PMC4895020; doi:10.1038/ncomms11696)
Supplement: Supplementary Information — Supplementary Figures 1-7 and Supplementary Table 1-2 [file ncomms11696-s1.pdf]

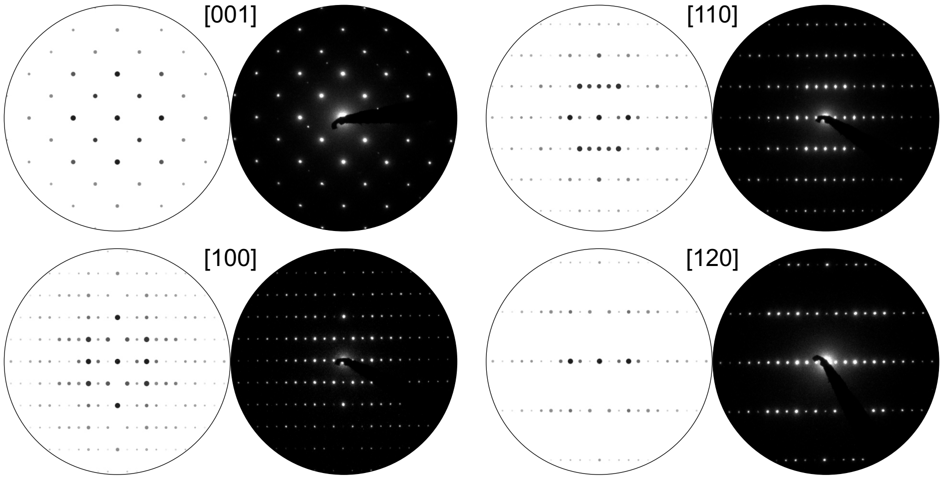

Supplementary Figure 1. Precession Electron Diffraction (PED) of ZrSiS along different zone axis. Simulated patterns are shown with a white background whereas measured patterns are shown with a black background. The match between the simulated and measured patterns is very good, confirming the single crystals diffraction results shown in Supplementary tables 1 and 2.

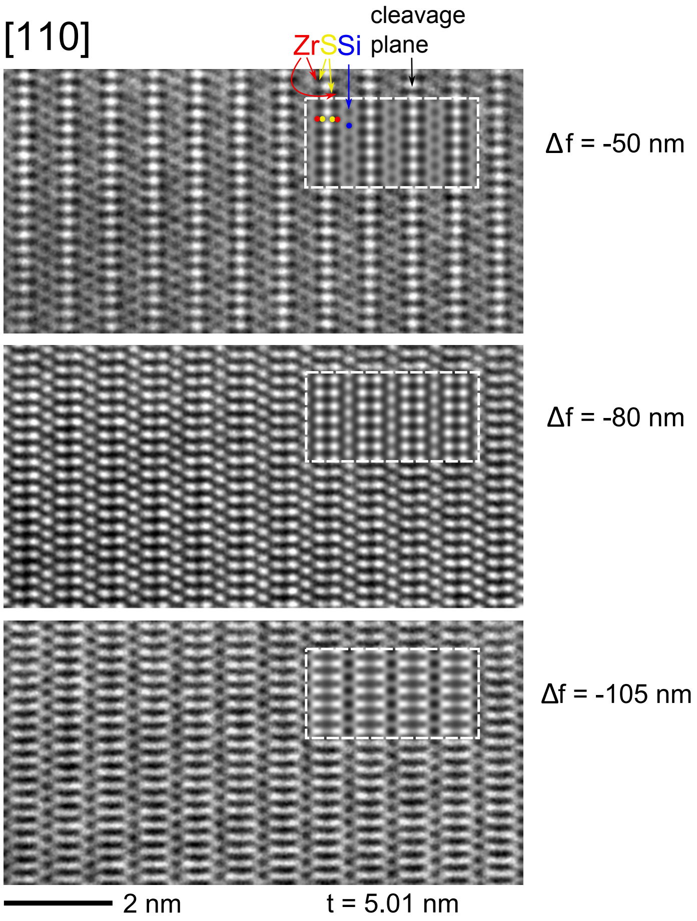

Supplementary Figure 2. HRTEM images of the (110) plane of ZrSiS, measured on a powdered sample. Top panel is close to Sherzer focus, where atoms appear in black and gaps in white. Individual atoms are labeled. Insets show simulated HRTEM images; simulations assume a sample thickness of t = 5.01nm.

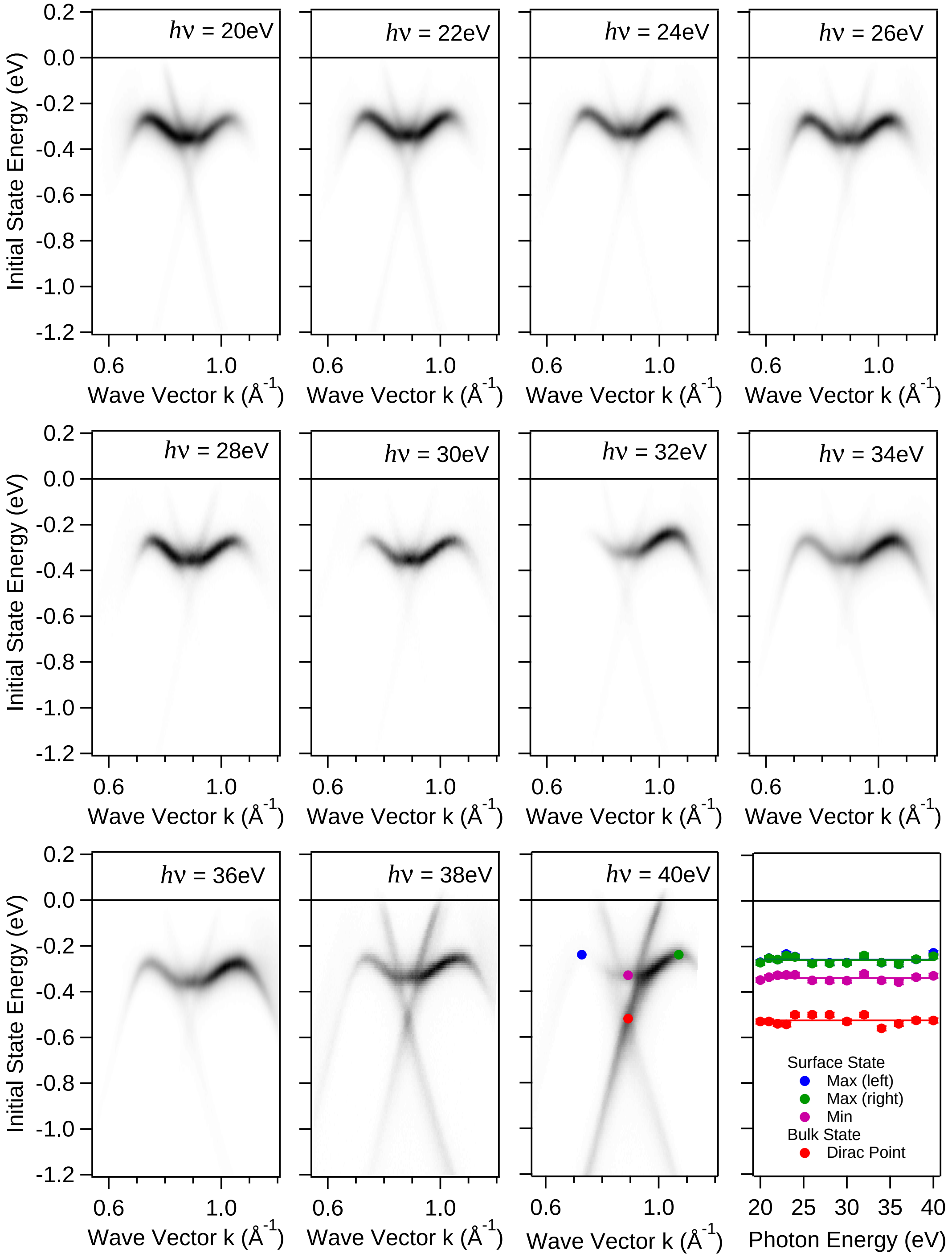

Supplementary Figure 3. ARPES spectra of ZrSiS near X along ΓX at different photon energies between 20 eV and 40 eV. The position of the bands does not change, as shown in the lower right panel for selected points in the band structure, supporting the two-dimensionality of the states. Changes in intensity are due to changing matrix elements as function of photon energy.

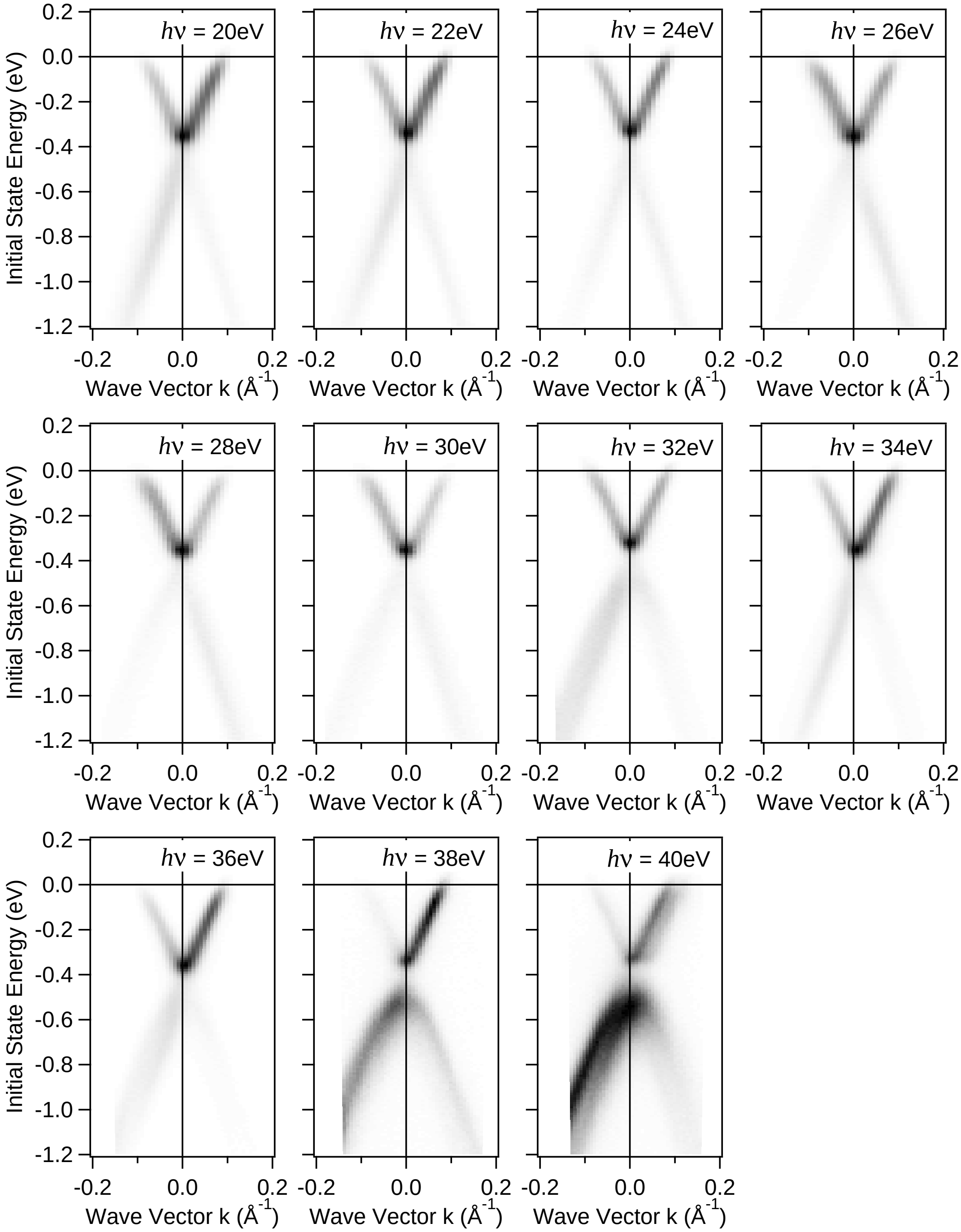

Supplementary Figure 4. ARPES spectra of ZrSiS near X along XM at different photon energies between 20 eV and 40 eV. The position of the bands does not change supporting the two-dimensionality of the states. Changes in intensity are due to changing matrix elements as function of photon energy.

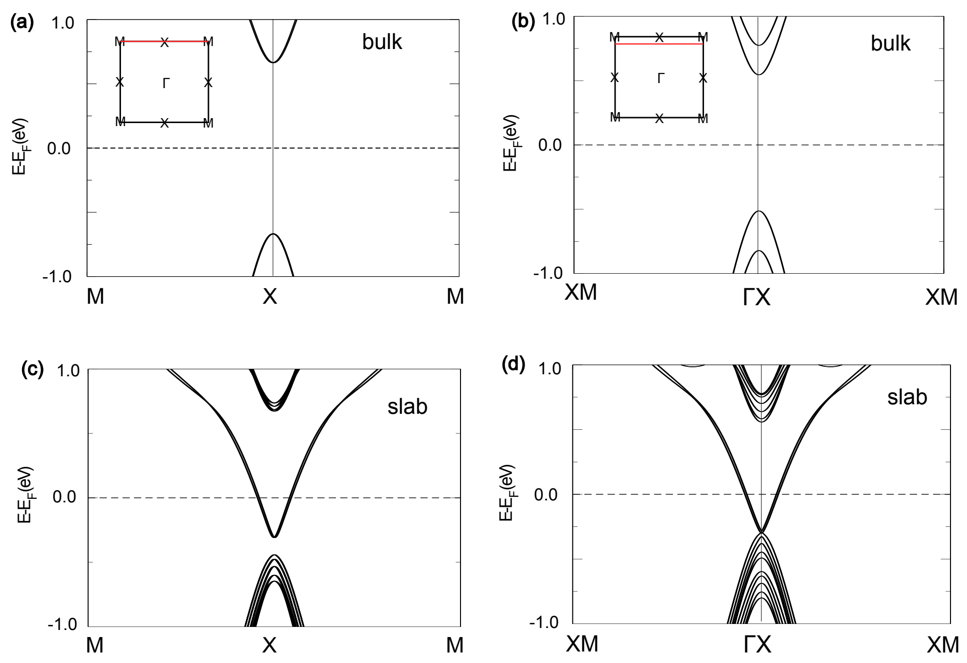

Supplementary Figure 5. Band structure along MX and parallel to MX. (a) and (b) show bulk band structures where the BZ is gapped along these directions, insets show the k path. (c) and (d) show slab band structures along the same paths, the gap is closed by surface states giving rise to a cone that is also observed in ARPES.

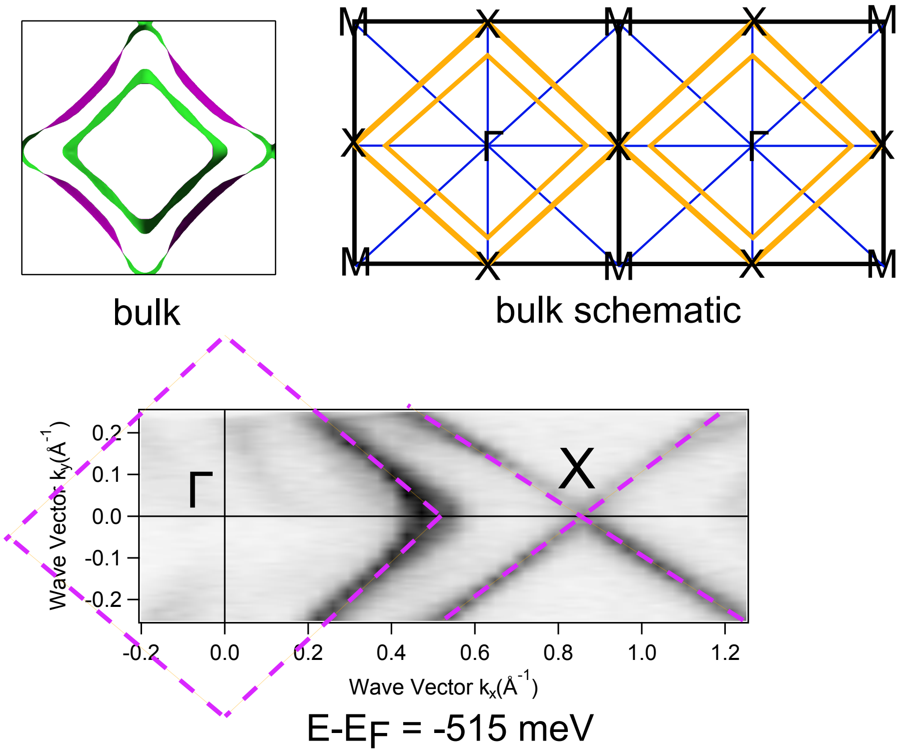

Supplementary Figure 6. Constant energy surface at E-E$_F$ = -515 meV. The energy surface now expands to the X point as expected. A second diamond shaped surface appears inside, which is the second branch of the bulk cones. The drawing on top sketches the predicted energy surface at -515 meV from bulk calculations (right), whereas the left shows the actually calculated bulk energy surface. Dashed purple lines indicate the measured bulk bands. Note that the crystal was not in a perfect 90 degree angle to the beam which is why the measured bands do not perfectly map the diamond shaped energy surface.

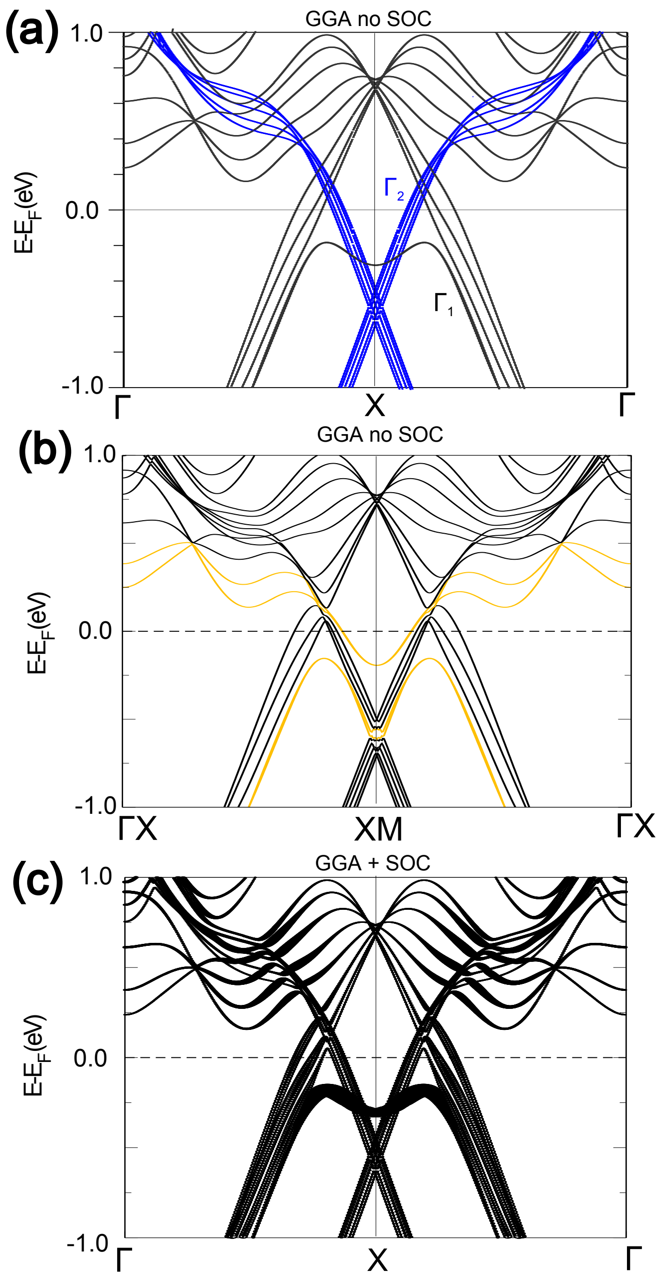

Supplementary Figure 7. Slab band structure along ΓX (a) calculated without spin orbit coupling showing that two different irreducible representations are possible and the surface state does not mix with the bulk bands. (b) Calculated without SOC and plotted parallel to ΓX where only one irreducible representation is allowed and hence mixing of the states is allowed, even without SOC. (c) Calculated with SOC but contribution of surface atoms is plotted thick, indicating clearly that the surface state arises from these atoms even if it is hybridized with the bulk.

Supplementary Table I. Crystallographic data and details of data collection.

Formula Sum	ZrSiS
Crystal system	Tetragonal
space group	$P4/nmm$ (No. 129)
Formula weight (g/mol)	151.37
Density (g/cm^3)	4.964
a (Å)	3.5450(5)
c (Å)	8.0578(16)
V (Å^3)	101.27(3)
Z	2
Temperature (K)	293(2)
Radiation	Mo K_α
2Θ range	10.13 - 63.36
F_{000}	140
Unique reflections	132
Difference e^- density (e/Å^3)	+0.917 to -1.061
Extinction coefficient	0.087(11)
R_1 (all reflections)	0.0205
R_1 ($F_0 > 4\sigma(F_0)$)	0.0153
wR_2	0.0375
Absorption coefficient μ (mm^{-1})	3.27
GooF	1.18

Supplementary Table II. Position coordinates and thermal displacement parameters for ZrSiS

Atom	Wyck	x	y	z	Occ.	U_{11} [Å^2]	U_{22} [Å^2]	U_{33} [Å^2]
Zr	2c	0.25000	0.25000	0.72906(6)	1	0.0048(2)	0.0048(2)	0.0052(3)
S	2c	0.25000	0.25000	0.37915(15)	1	0.0057(4)	0.0057(4)	0.00715(5)
Si	2a	0.25000	-0.25000	0.0000	1	0.0060(4)	0.0060(4)	0.0062(6)
